# Survival Outcomes of Gemcitabine–Cisplatin–S‐1 Versus Gemcitabine–Cisplatin in Unresectable Biliary Tract Cancer: A Multicenter Retrospective Study With a Focus on Conversion Surgery

**DOI:** 10.1002/ags3.70202

**Published:** 2026-02-20

**Authors:** Hisashi Kosaka, Masahiko Kinoshita, Satoshi Yasuda, Satoru Kakizaki, Rie Sugimoto, Jun Sakata, Kiyonori Yamai, Takeshi Hatanaka, Yusuke Yamamoto, Toshifumi Sato, Toru Ishikawa, Takanori Morikawa, Jun Hanaoka, Haruki Mori, Hidenori Toyoda, Tsuyoshi Sanuki, Misaki Yokoi, Hiroyuki Shibata, Koji Fukuda, Kazuhito Kawata, Koji Amaya, Takashi Ito, Masaaki Hidaka, Atsushi Naganuma, Keishi Sugimachi, Satoshi Toshiyama, Makoto Yamasaki, Masaki Kaibori

**Affiliations:** ^1^ Department of Hepatobiliary Surgery Kansai Medical University Hirakata Japan; ^2^ Department of Hepatobiliary–Pancreatic Surgery Osaka Metropolitan University Osaka Japan; ^3^ Department of Surgery Nara Medical University Kashihara Japan; ^4^ Department of Clinical Research NHO Takasaki General Medical Center Takasaki City Japan; ^5^ Department of Hepato‐Biliary‐Pancreatology NHO Kyushu Cancer Center Fukuoka Japan; ^6^ Division of Digestive and General Surgery Niigata University Graduate School of Medical and Dental Sciences Niigata City Japan; ^7^ Department of Gastroenterology, Hematology and Oncology Odate Municipal General Hospital Odate City Japan; ^8^ Department of Gastroenterology Gunma Saiseikai Maebashi Hospital Maebashi Japan; ^9^ Department of Surgery Kyoto Prefectural University of Medicine Kyoto Japan; ^10^ Department of Gastroenterology Saiseikai Niigata Hospital Niigata Japan; ^11^ Department of Surgery Miyagi Cancer Center Natori City Japan; ^12^ Department of Gastroenterological Surgery Ehime Prefectural Central Hospital Matsuyama City Japan; ^13^ Department of Surgery Shiga University of Medical Science Otsu City Japan; ^14^ Department of Gastroenterology and Hepatology Ogaki Municipal Hospital Ogaki City Japan; ^15^ Department of Gastroenterology Hyogo Prefectural Harima‐Himeji General Medical Center Himeji City Japan; ^16^ Department of Clinical Oncology Akita University Graduate School of Medicine Akita City Japan; ^17^ Hepatology Division, Department of Internal Medicine II Hamamatsu University School of Medicine Hamamatsu City Japan; ^18^ Department of Surgery Toyama Prefectural Central Hospital Toyama Japan; ^19^ Third Department of Internal Medicine, Division of Gastroenterology and Hepatology Kansai Medical University Osaka Japan; ^20^ Digestive and General Surgery Shimane University Faculty of Medicine Matsue Japan; ^21^ Department of Gastroenterology NHO Takasaki General Medical Center Takasaki Japan; ^22^ Department of Hepatobiliary and Pancreatic Surgery NHO Kyushu Cancer Center Fukuoka Japan; ^23^ Department of Upper Gastrointestinal Surgery Kansai Medical University Moriguchi City Japan

**Keywords:** biliary tract neoplasms, cholangiocarcinoma, cisplatin, gemcitabine, Tegafur Gimeracil Oteracil potassium (S‐1)

## Abstract

**Background:**

Gemcitabine plus cisplatin (GC) has been the global standard for advanced biliary tract cancer (BTC). The triplet regimen gemcitabine–cisplatin–S‐1 (GCS) demonstrated superiority in the MITSUBA trial, but its real‐world effectiveness remains unclear. We compared survival outcomes of GCS versus GC, focusing on conversion surgery (CS).

**Methods:**

We retrospectively analyzed 542 patients with unresectable BTC treated between 2017 and 2024 at 19 Japanese institutions. Patients received GC (*n* = 310) or GCS (*n* = 232). Survival was evaluated using multivariable Cox regression, 90‐day landmark analysis, and propensity score matching (PSM) to adjust for baseline imbalances.

**Results:**

Patients treated with GCS achieved greater tumor shrinkage (median −23.0% vs. –10.0%, *p* = 0.014) and a higher CS rate (16.4% vs. 4.5%, *p* < 0.001) than GC. Median progression‐free survival was 8.6 versus 5.4 months (*p* = 0.002), and median overall survival (OS) was 17.2 versus 11.6 months (*p* = 0.006). In multivariable analysis, GCS was associated with a lower risk of death (HR 0.80, 95% CI 0.65–0.98, *p* = 0.035), with consistent results after PSM. Fifty‐two patients underwent CS, with comparable perioperative safety and R0 resection rates between regimens. Patients who underwent CS achieved markedly longer OS; in the overall cohort, median OS was 31.0 months in the GCS with CS group and not reached in the GC with CS group (*p* = 0.131).

**Conclusions:**

Treatment with GCS was associated with longer survival compared with GC in unresectable BTC, alongside a higher rate of conversion surgery, which was associated with favorable long‐term outcomes.

## Graphical abstract

In this multicenter retrospective study conducted by the Biliary Tract Club, we compared survival outcomes between gemcitabine–cisplatin–S‐1 (GCS) and gemcitabine–cisplatin (GC) in patients with unresectable biliary tract cancer, with a particular focus on conversion surgery. GCS was associated with longer overall and progression‐free survival compared with GC across multiple analytical approaches, while conversion surgery—achieved in selected patients—was independently associated with favorable survival outcomes irrespective of the chemotherapy regimen. These findings support the clinical relevance of triplet chemotherapy and underscore the prognostic importance of conversion surgery within multidisciplinary treatment strategies for unresectable biliary tract cancer.

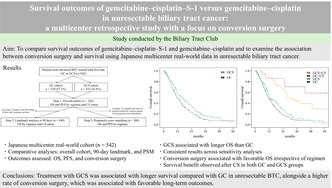

## 1 Introduction

Biliary tract cancer (BTC) remains a highly lethal malignancy, with the majority of patients presenting with advanced or unresectable disease at diagnosis [1]. Since the ABC‐02 trial, gemcitabine plus cisplatin (GC) has been established as the global standard first‐line chemotherapy for advanced BTC [2]. Nevertheless, the survival benefit of GC is modest, and more effective strategies are required. To address this limitation, alternative regimens have been explored in Japan. The phase III JCOG1113 (FUGA‐BT) trial demonstrated the non‐inferiority of gemcitabine plus S‐1 (GS) compared with GC in terms of overall survival (OS), thereby establishing GS as a standard option in Japanese practice [3]. Building on this evidence, the triplet regimen gemcitabine–cisplatin–S‐1 (GCS) was subsequently developed, and the phase III KHBO1401‐MITSUBA trial showed that GCS achieved a higher response rate than GC and translated this into a statistically significant survival advantage, supporting its role as a promising first‐line option for advanced BTC [4].

A recent meta‐analysis demonstrated that conversion surgery (CS) is associated with favorable survival in patients with initially unresectable BTC [5]. Consistently, an ancillary study of the KHBO1401 trial showed that GCS enabled CS with promising outcomes [6], and we recently reported a single‐center analysis confirming the survival benefit of GCS followed by CS [7]. However, although the KHBO1401‐MITSUBA trial established the superiority of GCS over GC under clinical trial conditions, it remains unclear whether these favorable results can be reproduced in real‐world practice, where patient backgrounds and treatment feasibility are more heterogeneous.

Therefore, we conducted a multicenter retrospective study to compare the effectiveness of GCS versus GC as first‐line chemotherapy for unresectable BTC in real‐world practice, focusing on survival outcomes and the feasibility of conversion surgery.

## 2 Materials and Methods

### 2.1 Patients

**FIGURE 1:**
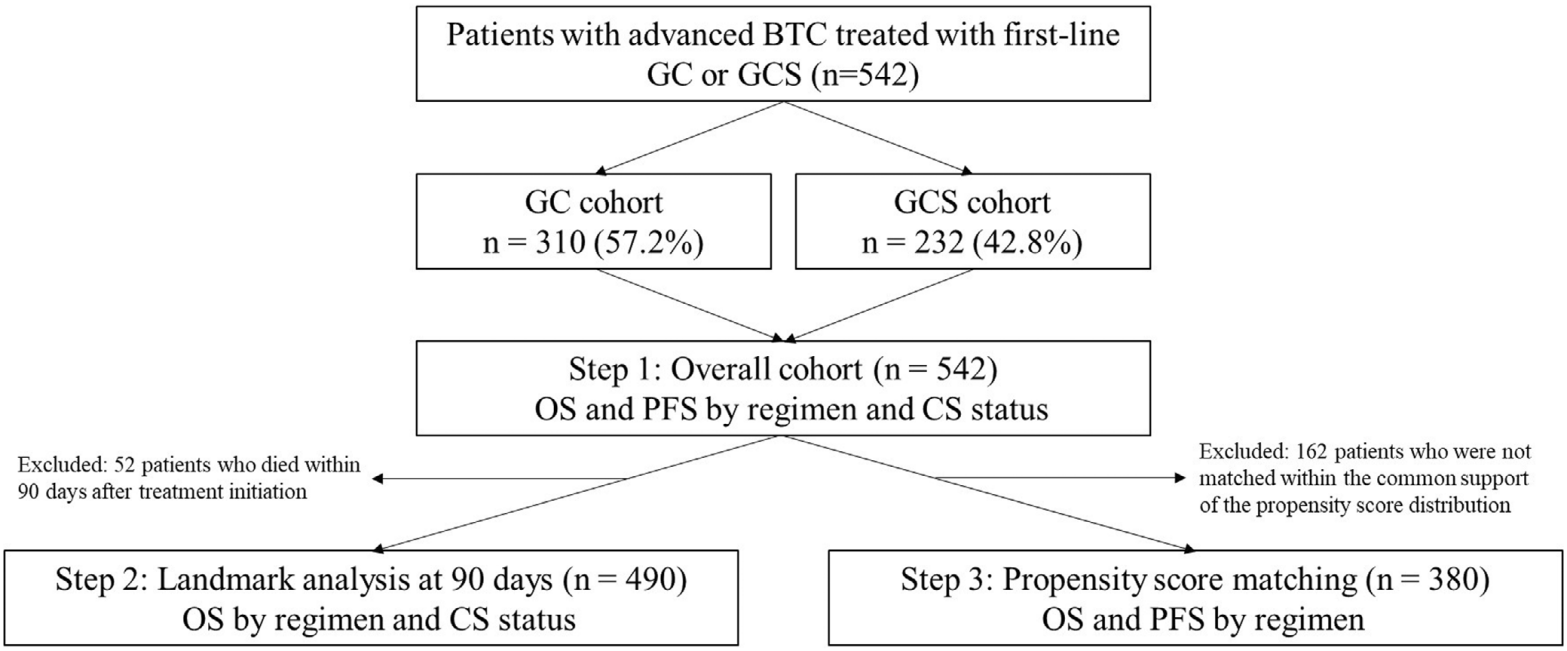
Study design and analytical flow. Patients with advanced biliary tract cancer treated with first‐line GC or GCS were included. Analyses were conducted in the overall cohort, a 90‐day landmark cohort, and a propensity score‐matched cohort. BTC, biliary tract cancer; GC, gemcitabine plus cisplatin; GCS, gemcitabine, cisplatin, and S‐1; OS, overall survival; PFS, progression‐free survival; CS, conversion surgery.

This multicenter retrospective study included patients who received first‐line systemic chemotherapy for unresectable or recurrent BTC between 2017 and 2024 at 19 institutions in Japan. Unresectability was determined by a multidisciplinary team at each institution based on radiological and clinical findings. A total of 542 patients were included in the analysis: 310 who received GC and 232 who received GCS (Figure 1). Baseline characteristics were summarized in Table 1. Clinical information, laboratory data, treatment details, and follow‐up outcomes were retrospectively collected from the medical records of each participating institution. This study was approved by the Institutional Review Board of Kansai Medical University (approval no. 2023320) and conducted within the framework of the Biliary Tract Club. The study was conducted in accordance with the principles of the Declaration of Helsinki.

**TABLE 1:** Baseline characteristics of the patients with unresectable biliary tract cancers.

Parameters	GC (*n* = 310)	GCS (*n* = 232)	*p*
Age	71.0 (65.0–76.0)	71.0 (63.3–76.0)	0.710
Gender, male	189 (61.0)	141 (60.8)	0.964
ECOG PS, 0–1	291 (93.9)	213 (91.8)	0.353
Primary tumor site			< 0.001
PHC	75 (24.2)	84 (36.2)	
ICC	90 (29.0)	77 (33.2)	
GBC	67 (21.6)	46 (19.8)	
DCC	56 (18.1)	20 (8.6)	
AC	22 (7.1)	5 (2.2)	
Recurrent tumor	127 (41.0)	68 (29.3)	0.005
Distant metastasis	209 (67.4)	123 (53.0)	0.001
Biliary drainage	97 (31.3)	103 (44.4)	0.002
Total bilirubin, mg/dL	0.7 (0.5–1.1)	0.8 (0.5–1.2)	0.139
Albumin, g/dL	3.6 (3.2–4.0)	3.7 (3.3–4.0)	0.248
Platelet count, ×10^4^/μL	22.3 (16.49–28.8)	22.3 (16.4–28.8)	0.747
CRP, mg/dL	0.46 (0.14–2.08)	0.48 (0.18–1.88)	0.823
ALBI score	−2.36 (−2.70 to −1.88)	−2.40 (−2.71 to −1.95)	0.405
CA19‐9, U/mL	136.8 (24.0–1243.1)	188.4 (25.8–1374.0)	0.679
Treatment initiation year	2021 (2020–2022)	2020 (2019–2022)	< 0.001

*Note:* Values are expressed as number (%) or median (interquartile range). *p* values were calculated using the chi‐squared test or Mann–Whitney *U* test, as appropriate.

Abbreviations: AC, ampullary cancer; ALBI, albumin–bilirubin; CA19‐9, carbohydrate antigen 19‐9; CRP, C‐reactive protein; DCC, distal cholangiocarcinoma; ECOG PS, Eastern Cooperative Oncology Group performance status; GBC, gallbladder cancer; GC, gemcitabine plus cisplatin; GCS, gemcitabine plus cisplatin and S‐1; ICC, intrahepatic cholangiocarcinoma; PHC, perihilar cholangiocarcinoma.

### 2.2 Definitions of Unresectable Biliary Tract Cancer and Conversion Surgery

Unresectability was determined by a multidisciplinary team at each institution based on radiological and clinical findings. In general, unresectable disease was defined according to technical, functional, and oncological criteria. Technical criteria included unreconstructable biliary or major vascular involvement precluding curative resection. Functional criteria referred to insufficient future liver remnant, poor hepatic reserve, or impaired performance status, with institutional thresholds for ICG‐Krem varying slightly (0.05–0.06) [8]. In the present cohort, insufficient future liver remnant was identified as a contributing factor for unresectability in 37 of the 542 patients (6.8%). Oncological criteria comprised the presence of distant metastasis.

CS was defined as curative‐intent resection performed when unresectability factors were improved to the extent that curative resection was considered feasible following systemic chemotherapy. There was no predefined minimum duration of chemotherapy for CS, and surgical candidacy was assessed whenever improvement of unresectable factors was confirmed on radiological reassessment. Decisions regarding CS were not based solely on the degree of tumor shrinkage; rather, sufficient improvement of unresectability factors was considered essential for surgical eligibility. Vascular resection and reconstruction were performed when R0 resection was considered feasible, and the final indication for CS was determined through multidisciplinary discussion at each institution.

### 2.3 Chemotherapy Regimens

The GC regimen consisted of gemcitabine (1000 mg/m^2^) and cisplatin (25 mg/m^2^) administered intravenously on Days 1 and 8 of a 21‐day cycle. The GCS regimen was administered according to the phase III KHBO1401‐MITSUBA protocol [4]. Gemcitabine (1000 mg/m^2^) and cisplatin (25 mg/m^2^) were given intravenously on Day 1 of a 14‐day cycle, combined with oral S‐1 administered twice daily on Days 1–7. The daily dose of S‐1 was determined according to body surface area (BSA): 80 mg/day for BSA < 1.25 m^2^, 100 mg/day for BSA 1.25–1.50 m^2^, and 120 mg/day for BSA ≥ 1.50 m^2^, administered in two divided doses. Dose reductions and schedule modifications were implemented at each institution according to patient condition and treatment tolerance.

### 2.4 Assessment

OS was defined as the time from initiation of first‐line chemotherapy to death from any cause or last follow‐up, and PFS as the time from treatment initiation to disease progression or death. Tumor response was assessed by Response Evaluation Criteria in Solid Tumors (RECIST) version 1.1, adverse events by the Common Terminology Criteria for Adverse Events (CTCAE) version 5.0, postoperative complications by the Clavien–Dindo classification, histological response by Evans' classification. Staging of intrahepatic cholangiocarcinoma was assessed according to the Japanese Classification of Liver Cancer (6th edition). Extrahepatic BTCs were assessed according to the Japanese Classification of Biliary Tract Carcinoma (7th edition); however, as no system‐specific sub‐classifications were applied, staging of extrahepatic BTCs was effectively based on the UICC TNM classification (8th edition).

### 2.5 Statistical Analysis

Categorical variables were summarized as numbers with percentages, and continuous variables as medians with interquartile ranges (IQRs), as appropriate. Categorical variables were compared using the *χ* ^2^ test or Fisher's exact test, and continuous variables using the Mann–Whitney *U* test. OS and progression‐free survival (PFS) were estimated using the Kaplan–Meier method and compared with the log‐rank test. Univariable Cox proportional hazards analyses were first performed to evaluate the association between each clinicopathological variable and OS. Multivariable Cox proportional hazards regression was performed to identify prognostic factors for OS, with results expressed as hazard ratios (HRs) and 95% confidence intervals (CIs). Covariates for the multivariable model were selected based on clinical relevance and prior evidence, with particular reference to the stratification factors used in the KHBO1401‐MITSUBA trial (primary tumor site, recurrent vs. initially unresectable disease, and presence or absence of distant metastasis). Additional prognostically relevant variables were included while avoiding model overfitting [4]. To reduce immortal time bias, a landmark analysis was performed with Day 90 from initiation of first‐line chemotherapy as the landmark time point. Only patients who were alive and under observation at Day 90 were included in the landmark analysis. The 90‐day landmark was selected as it corresponds to a clinically meaningful early treatment period during which initial therapeutic response is commonly assessed in routine clinical practice. Furthermore, to evaluate the independent impact of conversion surgery on overall survival while accounting for potential immortal time bias, a multivariable Cox proportional hazards model incorporating conversion surgery as a time‐dependent covariate was constructed using a start–stop (counting process) approach. In addition, propensity score matching (PSM) was performed to adjust for baseline imbalances between the treatment groups. Propensity scores were estimated using a logistic regression model including age, sex, Eastern Cooperative Oncology Group performance status, primary tumor site, recurrent versus initially unresectable disease, distant metastasis, biliary drainage, baseline laboratory variables (total bilirubin, albumin, platelet count, C‐reactive protein, and CA19‐9), and treatment initiation year. One‐to‐one nearest‐neighbor matching without replacement was conducted using a caliper width of 0.2 times the standard deviation of the logit of the propensity score, and patients outside the common support were excluded. Covariate balance after matching was assessed using standardized mean differences, with values < 0.10 considered acceptable. Survival analyses in the matched cohort were performed using Kaplan–Meier methods and Cox proportional hazards models.

PSM analyses were performed using R software, and all other statistical analyses were conducted using IBM SPSS Statistics for Windows, version 22.0 (IBM Japan Ltd., Tokyo, Japan). A two‐sided *p* < 0.05 was considered statistically significant.

## 3 Results

### 3.1 Patient Characteristics

A total of 542 patients were included in the present study, comprising 310 in the GC group and 232 in the GCS group (Table 1). The median age was 71 years in both groups, and the distribution of sex and ECOG performance status was comparable. Regarding primary tumor site, significant differences were observed between the groups (*p* < 0.001): perihilar cholangiocarcinoma and intrahepatic cholangiocarcinoma were more frequent in the GCS group, whereas distal cholangiocarcinoma and ampullary cancer were more common in the GC group. Recurrent disease was less frequent in the GCS group compared with the GC group (29.3% vs. 41.0%, *p* = 0.005). Details of recurrent tumor sites among patients with recurrence were summarized in Table S1. The proportion of patients with distant metastasis was also lower in the GCS group (53.0% vs. 67.4%, *p* = 0.001). Details of distant metastatic organs among patients with distant metastasis were summarized in Table S2. In contrast, biliary drainage was more frequently performed in the GCS group (44.4% vs. 31.3%, *p* = 0.002). Baseline laboratory data, including serum bilirubin, albumin, platelet count, C‐reactive protein, ALBI score, and CA19‐9 levels, were comparable between the two groups.

### 3.2 Treatment Response

Best overall response by RECIST version 1.1 was summarized in Table 2. The distribution of response categories differed significantly between the two regimens (*p* < 0.001). Complete response was rare in both groups (1.9% in GC and 2.2% in GCS), whereas partial response was observed more frequently in the GCS group than in the GC group (18.1% vs. 11.3%). The proportion of patients with stable disease and progressive disease was similar between the groups. When not evaluable cases were excluded, the objective response rate was 21.1% in the GCS group and 15.5% in the GC group, without a statistically significant difference (*p* = 0.113). The disease control rate was also comparable (70.4% vs. 68.2%, *p* = 0.597). The median best tumor shrinkage was significantly greater in the GCS group than in the GC group (−23.0% [IQR −40.0 to 0.0] vs. –10.0% [IQR −35.3 to 0.0], *p* = 0.014). Importantly, the proportion of patients who underwent CS was significantly higher in the GCS group than in the GC group (16.4% vs. 4.5%, *p* < 0.001).

**TABLE 2:** Best overall response by RECIST 1.1 according to treatment regimen.

Outcomes	GC (*n* = 310)	GCS (*n* = 232)	*p*
RECIST, best response			< 0.001
CR	6 (1.9)	5 (2.2)	
PR	35 (11.3)	42 (18.1)	
SD	139 (44.8)	110 (47.4)	
PD	84 (27.1)	66 (28.4)	
NE	46 (14.8)	9 (3.9)	
ORR (CR + PR)	41/264 (15.5)	47/223 (21.1)	0.113
DCR (CR + PR + SD)	180/264 (68.2)	157/223 (70.4)	0.597
Best tumor shrinkage, %	−10.0 (−35.3 to 0.0)	−23.0 (−40.0 to 0.0)	0.014
Conversion surgery	14 (4.5)	38 (16.4)	< 0.001

*Note:* Values are presented as number (%) or median (interquartile range). *p* values were calculated using the chi‐square test, Fisher's exact test, or the Mann–Whitney *U* test, as appropriate. Percentages for each response category were calculated with the total number of patients in each group as the denominator. Objective response rate and disease control rate were calculated among evaluable patients, excluding not evaluable cases from the denominator. Best tumor shrinkage represents the best percentage change in tumor size from baseline, calculated among patients achieving complete response, partial response, or stable disease, excluding not evaluable cases.

Abbreviations: CR, complete response; DCR, disease control rate; GC, gemcitabine plus cisplatin; GCS, gemcitabine, cisplatin, and S‐1; NE, not evaluable; ORR, objective response rate; PD, progressive disease; PR, partial response; RECIST, Response Evaluation Criteria in Solid Tumors; SD, stable disease.

### 3.3 Survival Outcomes

**FIGURE 2:**
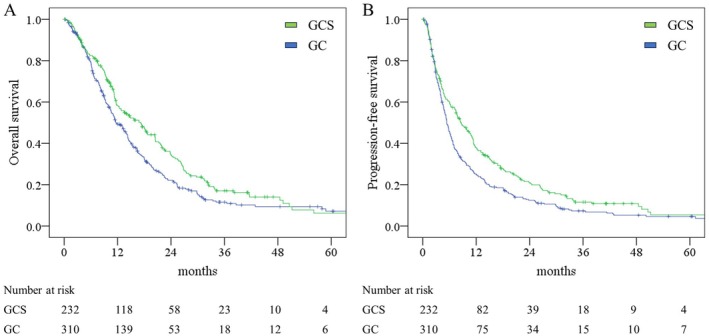
Overall survival and progression‐free survival according to treatment regimen. Kaplan–Meier curves for overall survival (A) and progression‐free survival (B) in patients with unresectable biliary tract cancer treated with gemcitabine plus cisplatin and S‐1 (GCS; Green line) or gemcitabine plus cisplatin (GC; Blue line). CI, confidence interval; GC, gemcitabine plus cisplatin; GCS, gemcitabine, cisplatin, and S‐1; mOS, median overall survival; mPFS, median progression‐free survival.

Kaplan–Meier curves for OS and PFS according to treatment regimen were shown in Figure 2. The median OS was significantly longer in the GCS group than in the GC group (17.2 vs. 11.6 months, *p* = 0.006). Similarly, the median PFS was significantly longer in the GCS group compared with the GC group (8.6 vs. 5.4 months, *p* = 0.002). In the multivariable Cox proportional hazards analysis (Table 3), treatment with GCS was associated with a lower risk of death compared with GC (HR 0.798, 95% CI 0.647–0.984, *p* = 0.035). In addition, gallbladder cancer, distant metastasis, higher ALBI score, and elevated CA19‐9 levels were associated with worse overall survival.

**TABLE 3:** Multivariable Cox proportional hazards analysis for overall survival.

Parameters	Univariable HR (95% CI)	*p*	Multivariable HR (95% CI)	*p*
Age	0.995 (0.985–1.005)	0.322	—	—
Gender, male	1.052 (0.954–1.160)	0.312	—	—
ECOG PS, 0–1	0.957 (0.674–1.360)	0.806	—	—
Primary tumor site				
PHC (Ref)	1.000	—	1.000	—
ICC	1.176 (0.919–1.505)	0.198	1.089 (0.905–1.310)	0.369
GBC	1.452 (1.104–1.909)	0.008	1.231 (1.002–1.511)	0.047
DCC	1.007 (0.725–1.397)	0.969	0.771 (0.603–0.987)	0.039
AC	1.392 (0.904–2.145)	0.133	1.037 (0.732–1.468)	0.840
Recurrent tumor (vs. primary)	0.920 (0.753–1.123)	0.411	1.088 (0.872–1.357)	0.454
Distant metastasis (vs. none)	1.348 (1.105–1.644)	0.003	1.382 (1.111–1.719)	0.004
ALBI	1.748 (1.523–2.006)	< 0.001	1.765 (1.531–2.034)	< 0.001
CA19‐9 (log‐transformed)	1.269 (1.167–1.379)	< 0.001	1.221 (1.119–1.331)	< 0.001
Treatment initiation year	0.965 (0.913–1.020)	0.211	1.013 (0.955–1.075)	0.663
GCS (vs GC)	0.761 (0.626–0.925)	0.006	0.798 (0.647–0.984)	0.035

*Note:* Hazard ratios with 95% confidence intervals are shown. Reference categories: PHC for primary tumor site, primary for recurrent tumor, none for distant metastasis, and GC for treatment group.

Abbreviations: AC, ampullary cancer; ALBI, albumin–bilirubin score; CA19‐9, carbohydrate antigen 19‐9; CI, confidence interval; DCC, distal cholangiocarcinoma; ECOG PS, Eastern Cooperative Oncology Group performance status; GBC, gallbladder cancer; GC, gemcitabine plus cisplatin; GCS, gemcitabine plus cisplatin and S‐1; HR, hazard ratio; ICC, intrahepatic cholangiocarcinoma; PHC, perihilar cholangiocarcinoma.

**FIGURE 3:**
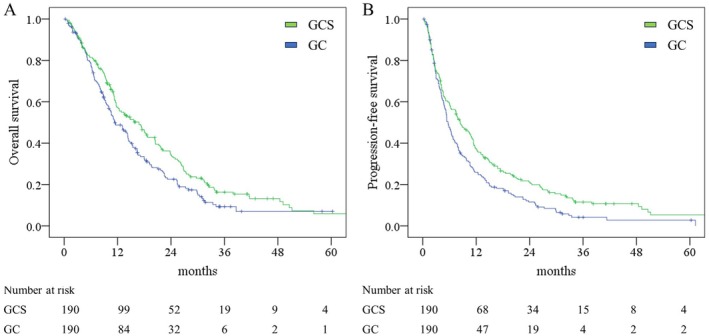
Overall survival and progression‐free survival after propensity score matching. Kaplan–Meier curves for overall survival (A) and progression‐free survival (B) in patients with unresectable biliary tract cancer treated with gemcitabine plus cisplatin and S‐1 (GCS; Green line) or gemcitabine plus cisplatin (GC; Blue line). Survival curves were compared using the log‐rank test in the propensity score–matched cohort. CI, confidence interval; GC, gemcitabine plus cisplatin; GCS, gemcitabine, cisplatin, and S‐1; mOS, median overall survival; mPFS, median progression‐free survival.

After PSM, baseline clinicopathological characteristics were well balanced between the GC and GCS groups, with all standardized mean differences below 0.10 (Table S3). The post‐matching Kaplan–Meier curves for OS and PFS were presented in Figure 3. OS remained significantly longer in the GCS group than in the GC group (16.8 vs. 11.3 months, *p* = 0.014). Progression‐free survival was also significantly longer with GCS (8.4 vs. 5.7 months, *p* = 0.004). Cox proportional hazards analyses were additionally performed in the matched cohort (Table S4). In the multivariable model, treatment with GCS remained associated with a reduced risk of death compared with GC. Higher ALBI score, elevated CA19‐9 levels, and the presence of distant metastasis were associated with worse overall survival. These findings were consistent with the results observed in the overall cohort.

### 3.4 Treatment‐Related Adverse Events

Treatment‐related adverse events of grade ≥ 3 were summarized in Table S5. Hematologic toxicities were more frequent in the GCS group than in the GC group. In particular, grade ≥ 3 neutropenia (31.5% vs. 19.0%, *p* = 0.001), anemia (11.6% vs. 5.2%, *p* = 0.006), and thrombocytopenia (12.1% vs. 3.5%, *p* < 0.001) occurred significantly more often in patients receiving GCS. In contrast, the incidence of non‐hematologic adverse events, including fatigue, decreased appetite, liver enzyme elevation, and cholangitis, did not differ significantly between the two regimens.

### 3.5 Conversion Surgery

A total of 52 patients underwent CS (GCS group: *n* = 38, 16.4%; GC group: *n* = 14, 4.5%), with a significantly higher proportion in the GCS group (*p* < 0.001). Patient characteristics and perioperative outcomes were summarized in Table 4. The median interval from treatment initiation to CS was similar between the groups (6.3 months in both). In the GCS group, major hepatectomy was performed more frequently, and the operative time was significantly longer compared with the GC group. However, the incidence of severe postoperative complications (Clavien–Dindo grade ≥ 3a) did not differ significantly between the two groups, and the 90‐day mortality rate was low in both groups. The R0 resection rate was comparable between the groups, and histological tumor regression, as assessed by Evans' grading, was similar between the two groups. Postoperative adjuvant chemotherapy was more frequently administered in the GCS group (76.3% vs. 50.0%), although the difference was not statistically significant.

**TABLE 4:** Details of conversion surgery.

Parameters	GC (*n* = 14)	GCS (*n* = 38)	*p*
Age at conversion, years	75.0 (67.5–80.0)	71.5 (65.8–75.5)	0.166
Gender, male	10 (71.4)	23 (60.5)	0.469
Interval to conversion, months	6.3 (5.0–8.9)	6.3 (4.0–7.5)	0.570
PTPE	1 (7.1)	7 (18.4)	0.423
Procedures			0.004
Major hepatectomy	4 (28.6)	22 (57.9)	
Minor hepatectomy	6 (42.9)	11 (28.9)	
Pancreatoduodenectomy	4 (28.6)	4 (10.5)	
Others	3 (21.4)	1 (2.6)	
Major vessel resection	1 (7.1)	5 (13.2)	0.670
Operative time, min	327.0 (235.8–486.5)	503.0 (401.5–631.0)	0.016
Blood loss, ml	518.0 (175.0–1170.5)	898.0 (602.5–1746.5)	0.069
Postoperative hospital stays, days	23.0 (9.8–55.5)	22.0 (10.5–38.5)	1.000
Clavien‐Dindo ≥ 3a	5 (36.7)	17 (44.7)	0.559
90‐days mortality	1 (7.1)	0 (0.0)	0.269
Histological Stage IV	9 (64.3)	16 (42.1)	0.156
Surgical margin status, R0/R1/R2	14/0/0 (100.0/0.0/0.0)	28/8/2 (73.7/21.1/5.3)	0.123
EVANS grade ≥ 2b	2/10 (20.0)	6/31 (19.4)	1.000
Adjuvant chemotherapy	7 (50.0)	29 (76.3)	0.094

*Note:* Values are presented as median (interquartile range) or *n* (%). *p* values were calculated using the *χ* ^2^ test or Fisher's exact test for categorical variables, and the Mann–Whitney *U* test for continuous variables, as appropriate. EVANS grade could be assessed in 10 patients in the GC group and 31 patients in the GCS group.

Abbreviations: GC, gemcitabine plus cisplatin; GCS, gemcitabine, cisplatin, and S‐1; HPD, hepatopancreatoduodenectomy; PTPE, preoperative transhepatic portal vein embolization.

**FIGURE 4:**
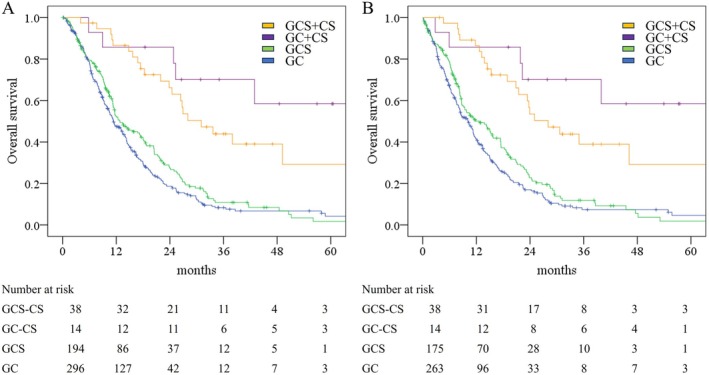
Kaplan–Meier curves of overall survival by treatment regimen and conversion surgery status. Kaplan–Meier curves for overall survival in patients with unresectable biliary tract cancer stratified by treatment regimen and conversion surgery status. Overall cohort (A) and landmark analysis including only patients who survived ≥ 90 days after initiation of first‐line chemotherapy (B). Patients were classified into four mutually exclusive groups according to treatment regimen and CS status: GCS with CS (orange), GC with CS (purple), GCS without CS (green), and GC without CS (blue). CI, confidence interval; CS, conversion surgery; GC, gemcitabine plus cisplatin; GCS, gemcitabine, cisplatin, and S‐1; mOS, median overall survival.

Kaplan–Meier analysis demonstrated that patients who underwent CS achieved substantially longer overall survival than those who did not, irrespective of treatment regimen (Figure 4). In the overall cohort, the median OS was 31.0 months in the GCS with CS, while it was not reached in the GC with CS group (*p* = 0.131). This survival advantage remained evident in the 90‐day landmark analysis (GCS with CS, 28.0 months vs. GC with CS, not reached, *p* = 0.132). In addition, 14 patients (26.9%) had recurrent BTC, and no significant difference in overall survival after conversion surgery was observed between patients with recurrent disease and those with initially unresectable disease.

To further evaluate the independent impact of conversion surgery on survival while accounting for potential immortal time bias, we additionally performed a multivariable Cox proportional hazards analysis incorporating conversion surgery as a time‐dependent covariate. In this model, conversion surgery was independently associated with improved overall survival, whereas the direct effect of GCS treatment was attenuated (Table S6).

## 4 Discussion

In this multicenter retrospective study, we found that the triplet regimen of GCS was associated with longer OS and PFS compared with GC in patients with unresectable BTC. Although baseline characteristics differed between the GC and GCS groups, PSM yielded results consistent with the primary analysis, further supporting the robustness of the observed associations after adjustment for measured confounders. These findings are generally consistent with the phase III KHBO1401‐MITSUBA trial, which established GCS as a more effective first‐line treatment option [4]. Importantly, our results extended the evidence for GCS to a real‐world setting, where patient backgrounds are more heterogeneous than in clinical trial cohorts. Notably, the median age of our cohort was 71 years, which is older than the 68 years reported in the MITSUBA trial, highlighting the potential applicability of GCS even in an elderly population. In terms of tumor response, GCS was associated with greater tumor shrinkage and was also associated with a higher CS rate than GC. This is consistent with the MITSUBA trial, which also demonstrated substantial tumor shrinkage with GCS, and our findings further confirm that this effect can be reproduced in routine clinical practice [4]. This is clinically meaningful because CS was associated with improved survival outcomes in our study. At the same time, these findings should be interpreted in the context of baseline differences between the treatment groups. The GC group included a higher proportion of patients with recurrent disease and distant metastasis, reflecting differences in disease status at treatment initiation, which may have influenced both survival outcomes and the feasibility of conversion surgery, independent of the treatment regimen itself. Although these imbalances were addressed using propensity score matching, the possibility of residual confounding cannot be completely excluded.

The survival benefit of CS has been increasingly recognized, and our findings are consistent with previous reports, including the Korea–Japan collaborative study [9] and early reports [10, 11], which mainly highlighted the prognostic value of doublet therapy followed by CS. We also previously reported a single‐center analysis suggesting the benefit of GCS followed by CS [7]. These observations are further supported by recent comprehensive reviews that have highlighted the evolving role of conversion surgery within multidisciplinary treatment strategies for unresectable BTC [12, 13]. Building on these findings, the present study is, to our knowledge, the first multicenter collaborative real‐world investigation to demonstrate an association between GCS followed by CS and favorable survival outcomes, thereby extending the evidence base for triplet chemotherapy in unresectable BTC. In our cohort, patients who underwent CS achieved markedly better OS than those who did not, regardless of the initial regimen. In the overall cohort, the median OS was 31.0 months in the GCS with CS group and was not reached in the GC with CS group (*p* = 0.131). Although not statistically significant, this finding suggests that prognosis may be favorable once CS is achieved. Importantly, because GCS was associated with greater tumor shrinkage than GC, a higher proportion of patients in the GCS group were able to undergo CS, which may represent one mechanism contributing to the observed survival differences. To further clarify the independent contribution of conversion surgery to survival while accounting for potential immortal time bias, we additionally performed a multivariable Cox proportional hazards analysis incorporating conversion surgery as a time‐dependent covariate. In this analysis, conversion surgery remained independently associated with improved overall survival, whereas the direct effect of GCS treatment was attenuated, suggesting that the survival benefit of GCS may be mediated, at least in part, through an increased likelihood of achieving conversion surgery.

The perioperative outcomes of CS were also notable. Despite longer operative times and more frequent major hepatectomies in the GCS group, the rates of severe postoperative complications and 90‐day mortality were comparable between regimens. Although the R0 resection rate was numerically lower in the GCS group without statistical significance, histological tumor regression by Evans' grading did not differ between groups, suggesting that perioperative safety and pathological outcomes were not clearly inferior in patients receiving GCS. However, these findings should be interpreted with caution given the limited sample size and potential differences in patient selection between treatment groups for conversion surgery. These findings are consistent with previous reports demonstrating that CS can be performed safely in selected patients with BTC [7, 9, 10, 11], further supporting the feasibility of integrating CS into treatment strategies following effective systemic therapy. Although the survival curve of the GCS plus CS subgroup appeared numerically inferior to that of the GC plus CS subgroup, this finding should not be interpreted as evidence of reduced antitumor efficacy of GCS. Importantly, the lack of a statistically significant difference in overall survival between the GC plus CS and GCS plus CS subgroups is more likely attributable to the limited sample size and insufficient statistical power of the conversion surgery subgroup, potentially compounded by differences in surgical complexity and patient selection among cases undergoing CS after GCS.

Finally, immune checkpoint inhibitors (ICIs) have recently been incorporated into first‐line therapy for BTC. The phase III TOPAZ‐1 trial demonstrated that durvalumab added to gemcitabine–cisplatin significantly improved survival compared with GC alone [14], and the KEYNOTE‐966 trial likewise showed a benefit with pembrolizumab plus GC [15]. These results have led to an increasing adoption of GC combined with ICIs in clinical practice. In addition, recent clinical studies have reported the feasibility of conversion surgery following response to ICI‐based combination therapy, with favorable survival outcomes observed in selected patients [16]. In this evolving landscape, further studies are warranted to clarify how triplet chemotherapy such as GCS should be optimally positioned—either as an independent regimen or in combination with ICIs—to define the most appropriate treatment strategies and patient selection.

## 5 Limitations

This study has several limitations. First, its retrospective design may have introduced selection bias, particularly regarding patient eligibility for CS. Second, the decision to perform CS was made at the discretion of each institution, and surgical indications or perioperative management were not standardized across centers. Third, baseline differences between the two groups, such as tumor site distribution, recurrence status, distant metastasis, and biliary drainage, may have influenced outcomes. To mitigate these imbalances, we performed multivariable Cox regression and newly incorporated propensity score matching, which consistently showed an association between GCS treatment and improved survival. Nonetheless, the possibility of residual confounding cannot be excluded. Finally, the follow‐up period and sample size, especially in the CS subgroup, may have limited the statistical power to detect differences in long‐term outcomes. These limitations should be considered when interpreting our findings.

## 6 Conclusions

In this multicenter retrospective study, GCS was associated with improved survival compared with GC in patients with unresectable BTC. GCS achieved greater tumor shrinkage and enabled conversion surgery in a higher proportion of patients, which might be linked to favorable prognosis. These findings suggest that GCS may represent a promising first‐line treatment option in unresectable biliary tract cancer, particularly through increasing the likelihood of achieving conversion surgery, although causal inference should be interpreted with caution given the retrospective study design.

## Author Contributions

**Hisashi Kosaka:** conceptualization, methodology, software, data curation, investigation, validation, formal analysis, supervision, visualization, writing – original draft, writing – review and editing. **Masahiko Kinoshita:** data curation. **Satoshi Yasuda:** data curation. **Satoru Kakizaki:** data curation. **Rie Sugimoto:** data curation. **Jun Sakata:** data curation. **Kiyonori Yamai:** data curation. **Takeshi Hatanaka:** data curation. **Yusuke Yamamoto:** data curation. **Toshifumi Sato:** data curation. **Toru Ishikawa:** data curation. **Takanori Morikawa:** data curation. **Jun Hanaoka:** data curation. **Haruki Mori:** data curation. **Hidenori Toyoda:** data curation. **Tsuyoshi Sanuki:** data curation. **Misaki Yokoi:** data curation. **Hiroyuki Shibata:** data curation. **Koji Fukuda:** data curation. **Kazuhito Kawata:** data curation. **Koji Amaya:** data curation. **Takashi Ito:** data curation. **Masaaki Hidaka:** data curation. **Atsushi Naganuma:** data curation. **Keishi Sugimachi:** data curation. **Satoshi Toshiyama:** supervision. **Makoto Yamasaki:** supervision. **Masaki Kaibori:** supervision.

## Funding

The authors have nothing to report.

## Ethics Statement

This study was approved by the Institutional Review Board of Kansai Medical University (Approval number: 2023320). It was performed in accordance with the Declaration of Helsinki.

## Conflicts of Interest

The authors declare no conflicts of interest.

## Supporting information


**Table S1:** Recurrent tumor sites in patients with recurrence.
**Table S2:** Distant metastatic organs in patients with distant metastasis.
**Table S3:** Baseline characteristics of patients with unresectable biliary tract cancer after propensity score matching.
**Table S4:** Multivariable Cox proportional hazards analysis for overall survival after propensity score matching.
**Table S5:** Treatment‐related adverse events (grade ≥ 3, by regimen).
**Table S6:** Multivariable Cox proportional hazards analysis for overall survival incorporating conversion surgery as a time‐dependent covariate.
