# Supplementary material for: Survival Outcomes of Gemcitabine–Cisplatin–S‐1 Versus Gemcitabine–Cisplatin in Unresectable Biliary Tract Cancer: A Multicenter Retrospective Study With a Focus on Conversion Surgery
Source: Ann Gastroenterol Surg. 2026 Feb 20;10(4):1261–70. doi: 10.1002/ags3.70202 (PMC13326820; doi:10.1002/ags3.70202)
Supplement: Supplementary file 1 — Table S1: Recurrent tumor sites in patients with recurrence. Table S2: Distant metastatic organs in patients with distant metastasis. Table S3: Baseline characteristics of patients with unresectable biliary tract cancer after propensity score matching. Table S4: Multivariable Cox proportional hazards analysis for overall survival after propensity score matching. Table S5: Treatment‐related adverse events (grade ≥ 3, by regimen). Table S6: Multivariable Cox proportional hazards analysis for overall survival incorporating conversion surgery as a time‐dependent covariate. [file AGS3-10-1261-s001.docx]

| **Supplemental Table 1. Recurrent tumor sites in patients with recurrence** | | | |
| --- | --- | --- | --- |
| *Parameters* | GC (n = 127) | GCS (n = 68) | *p* value |
| *Liver* | 44 (34.6) | 36 (52.9) | 0.013 |
| *Lymph node* | 27 (21.3) | 12 (17.6) | 0.548 |
| *Local recurrence* | 29 (22.8) | 6 (8.8) | 0.015 |
| *Peritoneum* | 21 (16.5) | 8 (11.8) | 0.372 |
| *Lung* | 17 (13.4) | 9 (13.2) | 0.976 |
| *Bone* | 3 (2.4) | 3 (4.4) | 0.430 |
| *Others* | 2 (1.6) | 1 (1.5) | 0.955 |
| Values are expressed as number (percentage). *p* values were calculated using the chi-square test. Patients with recurrence at multiple anatomic sites were counted in each corresponding category. Others included adrenal gland (n = 2) and pancreas (n = 1).  GC, gemcitabine plus cisplatin; GCS. | | | |

| **Supplemental Table 2. Distant metastatic organs in patients with distant metastasis** | | | |
| --- | --- | --- | --- |
| *Parameters* | GC (n = 209) | GCS (n = 123) | *p* value |
| *Liver* | 79 (37.8) | 52 (42.3) | 0.420 |
| *Distant lymph node* | 72 (34.4) | 37 (30.1) | 0.413 |
| *Peritoneum* | 49 (23.4) | 22 (17.9) | 0.233 |
| *Lung* | 45 (21.5) | 24 (19.5) | 0.662 |
| *Bone* | 16 (7.7) | 13 (10.6) | 0.364 |
| *Others* | 7 (3.3) | 2 (1.6) | 0.350 |
| Values are expressed as number (percentage). *p* values were calculated using the chi-square test. Patients with metastases at multiple anatomic sites were counted in each corresponding category.  Others included adrenal gland (n = 5), pancreas (n = 2), spleen (n = 1), and mesenteric root (n = 1). GC, gemcitabine plus cisplatin; GCS. | | | |

| **Supplementary Table 3. Baseline characteristics of patients with unresectable biliary tract cancer after propensity score matching** | | | |
| --- | --- | --- | --- |
| *Parameters* | GC (n = 190) | GCS (n = 190) | *SMD* |
| *Age* | 69.3 ± 9.9 | 68.8 ± 9.8 | 0.048 |
| *Gender, male* | 116 (61.1) | 114 (60.0) | 0.023 |
| *ECOG PS, 0-1* | 176 (92.6) | 176 (92.6) | <0.001 |
| *Primary tumor site* |  |  |  |
| *PHC* | 61 (32.1) | 64 (33.7) | 0.034 |
| *ICC* | 61 (32.1) | 63 (33.2) | 0.023 |
| *GBC* | 42 (22.1) | 41 (21.6) | 0.012 |
| *DCC* | 20 (10.5) | 17 (8.9) | 0.054 |
| *AC* | 6 (3.2) | 5 (2.6) | 0.036 |
| *Recurrent tumor* | 63 (33.2) | 59 (31.1) | 0.045 |
| *Distant metastasis* | 114 (60.0) | 114 (60.0) | <0.001 |
| *Biliary drainage* | 76 (40.0) | 79 (41.6) | 0.033 |
| *Total bilirubin, mg/dL* | 1.1 ± 1.1 | 1.0 ± 0.8 | 0.080 |
| *Albumin, g/dL* | 3.5 ± 0.6 | 3.6 ± 0.6 | 0.073 |
| *Platelet count, ×10^4^/μL* | 23.6 ± 11.0 | 23.5 ± 9.7 | 0.008 |
| *CRP, mg/dL* | 2.0 ± 3.6 | 2.0 ± 3.7 | 0.018 |
| *CA19-9, U/mL* | 11259.3 ± 93898.9 | 19135.4 ± 215861.7 | 0.047 |
| *Treatment initiation year* | 2020.8 ± 1.7 | 2020.9 ± 1.4 | 0.057 |
| Values are expressed as number (percentage) or mean ± standard deviation. Standardized mean differences (SMDs) < 0.10 were considered indicative of adequate covariate balance. The albumin–bilirubin (ALBI) score was not included as a covariate because its components (albumin and total bilirubin) were analyzed separately.  GC, gemcitabine plus cisplatin; GCS, gemcitabine plus cisplatin and S-1; ECOG PS, Eastern Cooperative Oncology Group performance status; PHC, perihilar cholangiocarcinoma; ICC, intrahepatic cholangiocarcinoma; GBC, gallbladder cancer; DCC, distal cholangiocarcinoma; AC, ampullary cancer; CRP, C-reactive protein; CA19-9, carbohydrate antigen 19-9. | | | |

| **Supplemental Table 4. Multivariable Cox proportional hazards analysis for overall survival after propensity score matching** | | | | |
| --- | --- | --- | --- | --- |
| *Parameters* | Univariable HR (95% CI) | *p* value | Multivariable HR (95% CI) | *p* value |
| *Age* | 0.997  (0.986 – 1.008) | 0.559 | – | – |
| *Gender, male* | 1.334  (1.059 – 1.682) | 0.015 | 1.131  (1.004 – 1.274) | 0.043 |
| *ECOG PS, 0–1* | 1.016  (0.679 – 1.520) | 0.938 | – | – |
| *Primary tumor site* |  |  |  |  |
| *PHC (Ref)* | 1.000 | – | 1.000 | – |
| *ICC* | 1.143  (0.864 – 1.513) | 0.348 | 1.014  (0.803 – 1.280) | 0.907 |
| *GBC* | 1.404  (1.033 – 1.908) | 0.030 | 1.079  (0.833 – 1.398) | 0.564 |
| *DCC* | 0.891  (0.566 – 1.403) | 0.618 | 0.742  (0.517 – 1.066) | 0.107 |
| *AC* | 1.309  (0.683 – 2.511) | 0.417 | 1.412  (0.837 – 2.383) | 0.196 |
| *Recurrent tumor (vs primary)* | 0.887  (0.694 – 1.134) | 0.339 | 1.080  (0.822 – 1.419) | 0.579 |
| *Distant metastasis (vs none)* | 1.388  (1.100 – 1.751) | 0.006 | 1.495  (1.156 – 1.933) | 0.002 |
| *ALBI* | 1.869  (1.568 – 2.228) | <0.001 | 1.920  (1.576 – 2.339) | <0.001 |
| *CA19-9 (log-transformed)* | 1.281  (1.160 – 1.415) | <0.001 | 1.170  (1.050 – 1.304) | 0.004 |
| *Treatment initiation year* | 0.981  (0.910 – 1.057) | 0.608 | 1.037  (0.961 – 1.120) | 0.350 |
| *GCS (vs GC)* | 0.751  (0.598 – 0.944) | 0.014 | 0.771  (0.611 – 0.974) | 0.029 |
| Hazard ratios with 95% confidence intervals are shown. Reference categories: PHC for primary tumor site, primary for recurrent tumor, none for distant metastasis, and GC for treatment group.  HR, hazard ratio; CI, confidence interval; ECOG PS, Eastern Cooperative Oncology Group performance status; PHC, perihilar cholangiocarcinoma; ICC, intrahepatic cholangiocarcinoma; GBC, gallbladder cancer; DCC, distal cholangiocarcinoma; AC, ampullary cancer; ALBI, albumin–bilirubin score; CA19-9, carbohydrate antigen 19-9; GCS, gemcitabine plus cisplatin and S-1; GC, gemcitabine plus cisplatin. | | | | |

| **Supplemental Table 5. Treatment-related adverse events (grade ≥3, by regimen)** | | | |
| --- | --- | --- | --- |
| *Parameters* | GC (n = 310) | GCS (n = 232) | *p* value |
| *Hematologic adverse events* |  |  |  |
| *Neutrophil count decreased* | 59 (19.0) | 73 (31.5) | 0.001 |
| *Hemoglobin decreased* | 16 (5.2) | 27 (11.6) | 0.006 |
| *Platelet count decreased* | 11 (3.5) | 28 (12.1) | <0.001 |
| *Non-hematologic adverse events* |  |  |  |
| *Fatigue* | 7 (2.3) | 11 (4.7) | 0.110 |
| *Decreased appetite* | 7 (2.3) | 11 (4.7) | 0.110 |
| *AST or ALT increased* | 1 (0.3) | 2 (0.9) | 0.579 |
| *Creatinine increased* | 3 (1.0) | 0 (0.0) | 0.264 |
| *Cholangitis* | 1 (0.3) | 1 (0.4) | 1.000 |
| *Others* | 7 (2.3) | 5 (2.2) | 0.936 |
| Values are presented as n (%). *p* values were calculated using the χ² test or Fisher’s exact test, as appropriate. GC, gemcitabine plus cisplatin; GCS, gemcitabine, cisplatin, and S-1; AST, aspartate aminotransferase; ALT, alanine aminotransferase. | | | |

| **Supplemental Table 6. Multivariable Cox proportional hazards analysis for overall survival incorporating conversion surgery as a time-dependent covariate** | | | | |
| --- | --- | --- | --- | --- |
| *Parameters* | Univariable HR (95% CI) | *p* value | Multivariable HR (95% CI) | *p* value |
| *Age* | 0.995  (0.986 – 1.005) | 0.328 | – | – |
| *Gender, male* | 1.105  (0.909 – 1.343) | 0.318 | – | – |
| *ECOG PS, 0–1* | 0.962  (0.677 – 1.367) | 0.828 | – | – |
| *Primary tumor site* |  |  |  |  |
| *PHC (Ref)* | 1.000 | – | 1.000 | – |
| *ICC* | 1.160  (0.907 – 1.485) | 0.238 | 1.140  (0.886 – 1.467) | 0.309 |
| *GBC* | 1.460  (1.110 – 1.920) | 0.007 | 1.296  (0.975 – 1.723) | 0.074 |
| *DCC* | 0.998  (0.719 – 1.385) | 0.990 | 0.905  (0.639 – 1.282) | 0.574 |
| *AC* | 1.397  (0.907 – 2.152) | 0.129 | 1.202  (0.754 – 1.918) | 0.440 |
| *Recurrent tumor (vs primary)* | 0.921  (0.754 – 1.124) | 0.418 | 1.029  (0.823 – 1.286) | 0.805 |
| *Distant metastasis (vs none)* | 1.344  (1.102 – 1.639) | 0.003 | 1.358  (1.094 – 1.686) | 0.006 |
| *ALBI* | 1.759  (1.534 – 2.017) | <0.001 | 1.668  (1.445 – 1.924) | <0.001 |
| *CA19-9 (log-transformed)* | 1.275  (1.173 – 1.386) | <0.001 | 1.218  (1.118 – 1.326) | <0.001 |
| *Treatment initiation year* | 0.968  (0.915 – 1.024) | 0.260 | 0.996  (0.938 – 1.057) | 0.888 |
| *GCS (vs GC)* | 0.767  (0.631 – 0.932) | 0.008 | 0.926  (0.748 – 1.146) | 0.477 |
| *Conversion surgery* | 0.297  (0.201 – 0.439) | <0.001 | 0.356  (0.238 – 0.533) | <0.001 |
| Hazard ratios with 95% confidence intervals are shown. Conversion surgery was modeled as a time-dependent covariate using a start–stop (counting process) approach. Reference categories: PHC for primary tumor site, primary for recurrent tumor, none for distant metastasis, and GC for treatment group.  HR, hazard ratio; CI, confidence interval; ECOG PS, Eastern Cooperative Oncology Group performance status; PHC, perihilar cholangiocarcinoma; ICC, intrahepatic cholangiocarcinoma; GBC, gallbladder cancer; DCC, distal cholangiocarcinoma; AC, ampullary cancer; ALBI, albumin–bilirubin score; CA19-9, carbohydrate antigen 19-9; GCS, gemcitabine plus cisplatin and S-1; GC, gemcitabine plus cisplatin. | | | | |
